# Estimation of Multi-Mycotoxin Contamination in South African Compound Feeds 

**DOI:** 10.3390/toxins4100836

**Published:** 2012-10-18

**Authors:** Patrick B. Njobeh, Mike F. Dutton, Annica Tevell Åberg, Per Haggblom

**Affiliations:** 1 Food, Environment and Health Research Group, Faculty of Health Science, University of Johannesburg, Doornfontein Campus, 2028 Gauteng, P.O. Box 17011, South Africa; Email: mdutton@uj.ac.za; 2 Department of Food Technology, Faculty of Science, University of Johannesburg, Doornfontein Campus, 2028 Gauteng, P.O. Box 17011, South Africa; 3 Department of Chemistry, Environment and Food Hygiene, National Veterinary Institute (SVA), Uppsala 75189, Sweden; Email: annica.aberg@sva.se (A.T.A.); per.haggblom@sva.se (P.H.); 4 Division of Analytical Pharmaceutical Chemistry, Uppsala University, Uppsala 75189, Sweden

**Keywords:** mycotoxins, fungi, feeds, health, South Africa

## Abstract

A total of 92 commercial compound feeds from South Africa were investigated for various mycotoxins. The data reveal the highest incidence of feed contamination for fumonisins (FB) (range: 104–2999 µg/kg) followed by deoxynivalenol (DON) (range: 124–2352 µg/kg) and zearalenone (ZEA) (range: 30–610 µg/kg). The incidence of ochratoxin A (OTA) and aflatoxins (AF)-contaminated samples were generally low, *i.e.*, 4% and 30% of samples with levels ranging between 6.4 and 17.1 µg/kg (mean: 9.9 µg/kg) for OTA and 0.2 to 71.8 µg/kg (mean: 9.0 µg/kg) for AF. No samples contained T-2 toxin or HT-2 toxin. However, all samples analyzed were contaminated with at least one mycotoxin with a majority containing several mycotoxins. In particular, 3 of 4 positive samples mainly cattle feeds that had relatively high contents of OTA (ranging from 7 to 17.1 µg/kg) also contained high amounts of AF (>27.5 µg/kg) together with FB, DON and ZEA. Apart from a few samples, the levels of mycotoxins may be regarded as safe for livestock production in South Africa. However, the persistent co-occurrence of mycotoxins in samples, especially those at high concentrations, *i.e.*, AF and OTA, together with other mycotoxins studied, may elicit synergistic or additive effects in animals. This should raise concern as multiple contaminations may pose a risk to livestock production and health.

## 1. Introduction

Mycotoxins are a class of low molecular weight compounds that are produced by toxigenic strains of moulds that infect food and feed commodities. They can cause serious animal health related problems as they may be hepatotoxic, nephrotoxic [1,2] and may impair immune responses resulting in increased risk to secondary infection [3]. In addition, these toxins also compromise animal performance arising from reduced feed intake, poor feed utilization, loss in weight gain, decreased barrier function, reduced reproductive capacity and even death [4] leading to serious economic losses [5]. 

Even though several mycotoxins have been detected in various commodities worldwide, those of greatest significance in Africa and other tropical countries are aflatoxins (AF) and fumonisins (FB) [6]. Literature also seems to suggest that in addition to the AF (AFB_1_, B_2_, G_1_ and G_2_) and FB (FB_1_, FB_2_ and FB_3_), ochratoxin A (OTA) and the trichothecenes (TH) are also significant. This is not only because of high toxicities of the compounds, but also that their significance rests mainly on the fact that levels above those currently regulated are often encountered in food and feed commodities. It is in such circumstances that monitoring for these mycotoxins in food and feed commodities before they go into the market is imperative, which does not reflect the case in most African countries.

Contamination of compound feeds by mycotoxins and the potential transfer of these toxins into animal products (egg, organs, meat and milk) for human consumption remain a major concern. The health effects of mycotoxins can be categorized as acute or chronic mycotoxicosis. Most exposures to mycotoxins are detrimental but this may vary from one animal species to the other as well as on the level and duration of exposure. In the Republic of South Africa, outbreaks of mycotoxicoses in animals have been reported due to consumption of contaminated feeds. There have been two episodes of aflatoxicosis reported among dogs, one in 1987 [7] and another in the early 2011 that left at least 220 dogs dead and several others affected in the Gauteng region [8]. Monitoring for mycotoxins in feeds or their raw materials is not routinely performed. Due to the recent event of aflatoxicosis outbreak, it has become necessary for the National Department of Agriculture (NDA), to pass a regulation mandating dairy feed and pet food producers to analyze for mycotoxins [9]. 

In this study, liquid chromatography tandem mass spectrometry (LC-MS/MS) and LC-fluorescence were used to screen for the 11 well known mycotoxins AFB_1_, B_2_, G_1_ and G_2_, FB_1_, FB_2_, OTA, DON, ZEA, T-2, and HT-2 in animal feeds from S. Africa. The aim of this report was to shed more light on the distribution patterns and co-occurrence of mycotoxins in feeds produced and marketed in the country. 

## 2. Materials and Methods

### 2.1. Sampling and Sample Preparation

Between October 2010 and February 2011, a total of 92 feed samples (about 1 kg each) for different classes of animals produced in 2010 and stored in the plant were donated by members of the Animal Feed Manufacturers Association (AFMA) of South Africa and sent to the Food, Environment and Health Research Group (FEHRG), University of Johannesburg. A list of the compound feeds sampled and their main components are presented in Table 1. The sampling protocol followed was that described by Reiter *et al*. [10]. Manual sampling was performed by taking incremental samples using a sampling spear, samples put in sealed plastic bags and sent to FEHRG where they were milled to pass through a 2 mm sieve. Subsequently, samples were thoroughly mixed and further split by coning and sub-samples put in sealed plastic bags and taken to the Department of Chemistry, Environment and Feed Hygiene, National Veterinary Institute, Uppsala, Sweden, where they were kept at 4 °C until analysis. 

**Table 1:** Major components of compound feed samples collected

Compound Feed	N ^a^	Major ingredients
Poultry	62	
Breeder	14	Cereals (mainly maize), maize germ, soya oilcake, sunflower oilcake
Broiler	28	Cereals (mainly maize), maize germ, soya oilcake, sunflower oilcake
Layer	20	Cereals (mainly maize), maize germ, soya oilcake, sunflower oilcake
Cattle ^b^	25	
Calf grower	8	Cereals (mainly maize), maize germ, cottonseed, soya oilcake, sunflower oilcake
Dairy	11	Cereals (mainly maize), maize germ, cottonseed, sunflower oilcake, Lucerne meal
Finisher	6	Cereals (mainly maize), maize germ, cottonseed, sunflower oilcake, Lucerne meal
Others	5	
Horse	3	Cereals (mainly maize), maize germ, full fat soya, Lucerne meal
Swine	2	Cereals (mainly maize), maize germ, soya oilcake, sunflower oilcake
**Total**	**92**	

^a^ Number of samples collected and analyzed.

^b^ Cotton seed used includes oilcake and full fat cotton estimated at inclusion levels of 5%, 15% & 20% for calf grower, dairy and finisher feeds, respectively.

### 2.2. Experimental

#### 2.2.1. Reagents and Apparatus

All chemicals used for mycotoxin extraction and quantification were of analytical grade. The water used in the study was purified using a Milli-Q purification system (>17 MΩ/cm, Millipore, Bedford, BA, USA). 

(a) Extraction and cleanup columns: MultiSep^®^226 (RomerLabs, Tulln, Austria) and AflaCLEAN™SELECT (LCTech GmbH, Dorfen, Germany).

(b) Extraction solvents:Acetonitrile:water:formic acid (74:25:1, v/v/v) (Solvent A); 80% acetonitrile (Solvent B). 

(c) Reference substances and preparations: Aflatoxins (B_1_, B_2_, G_1_ and G_2_), deoxynivalenol, fumonisins (B_1_ and B_2_), HT-2 toxin, T-2 toxin, ochratoxin A, zearalenone and meloxicam sodium salt (Sigma Aldrich, Steinheim, Germany). From mycotoxin standard stock solutions (stored at −20 °C), five working standard concentrations for calibration of the instruments and to establish external calibration plots were prepared in extraction solvent/water (1:1, v/v). Meloxicam was used as an internal standard (25 µL of a 100 ng/mL solution was added to 500 µL of both calibration solutions and samples) to control variation of the LC-MS/MS system between samples in long sample series, not to compensate for any extraction variations.

(d) Apparatus: Rotina 420R centrifuge (Hettich Lab Technologies, Tuttlingen, Germany); LC-MS/MS: Quaternary Surveyor HPLC system coupled to a TSQ Quantum Discovery triple quadrupole mass spectrometer with an electrospray ionization (ESI) source (Thermo Electron Corp., San José, CA, USA). The HPLC column (Synergi Hydro-RP of 4 µm particle size and 150 × 2.0 mm i.d) and the guard column (Security Guard cartridge 4.0 × 2.0 mm i.d) were both from Phenonenex (supplied by Scandinaviska Genetec, Västra Frölunda, Sweden); HPLC system from Dionex (now Thermo Fisher, Gothenburg, Sweden) equipped with a P680 HPLC pump and an RF2000 fluorescence detector. The HPLC column was a Grace Smart RP18, 5 µm particle size and 150 × 4.6 mm i.d (supplied by Sorbent AB, Västra Frölunda, Sweden).

(e) LC mobile phases: Water/methanol/1 M ammonium formate/formic acid (90:10:0.1:0.002, v/v/v/v) (Eluent A); methanol/water/1 M ammonium formate/formic acid (95:4.9:0.1:0.002, v/v/v/v) (Eluent B); and 0.15 g potassium bromide, 700 mL water, 100 µL nitric acid, 300 mL methanol and 200 mL acetonitril (Eluent C).

#### 2.2.2. Methods of Mycotoxin Analyses

##### 2.2.2.1. Dexynivalenol, Fumonisin B1, Fumonisin B2, HT-2, T-2, Ochratoxin and Zearalenone

An LC-MS/MS multi-mycotoxin method recently developed and in-house validated by Tevell Åberg *et al*. [11] was used for the simultaneous quantification of DON, FB_1_, FB_2_, HT-2, T-2, OTA and ZEA. In short, 10 g of sample were extracted with 50 mL Solvent A in an Ehrlenmeyer flask (horizontal shaking for 60 min). The content was transferred to a centrifuge tube and subsequently centrifuged for 10 min at 4000 rpm. 15 mL of the supernatant was defatted using 10 mL of hexane on a rotary shaker for 10 min. The hexane fraction was removed and discarded while the retained extract was divided into two fractions. For one fraction, 0.5 mL of the crude extract was transferred into a test tube containing 0.5 mL water and 25 µL of meloxicam (100 ng/mL), vortexed and afterward, centrifuged for 10 min at 4000 rpm. The content was transferred into a screw-capped vial and stored at 4 °C until analyzed for OTA, FB_1_ and FB_2_. From the other fraction, 5 mL of the crude extract was cleaned through a MultiSep^®^226 column (flow rate of 1 mL/min, assisted by vacuum) and then 0.5 mL of the cleaned extract was transferred into a test tube containing 0.5 mL water and 25 µL of meloxicam (100 ng/mL). The content was thoroughly mixed, transferred into a screw-capped vial and stored at 4 °C until quantification of DON, ZEA, T-2 and HT-2. All extracts were stored and analyzed within 24 h.

The chromatographic separation was achieved with gradient elution of Eluent A and B pumped at a flow rate of 200 µL/min. For DON, ZON, T-2 and HT-2, 100% A was delivered for 1.0 min, then B was gradually increased to 50% within 1.0 min and then held on 50% B for 1.0 min. Between 3.0 and 6.0 min, B was increased to 100%, and then held on 100% B for 7.0 min, followed by equilibration on 100% A between 13 and 17 min. For FB_1_, FB_2_ and OTA, the gradient was the same for the first 3.0 min, then B was increased to 100% between 3.0 and 7.0 min, then held on 100% B for 4.0 min, followed by equilibration on 100% A between 11.1 and 15.0 min. The injection volume of both the samples and the mycotoxin standard solutions was 10 µL. Detection and quantification of each mycotoxin was performed in the Selected Reaction Monitoring (SRM) mode, and for detailed conditions, see Table 2. 

**Table 2:** LC-MS/MS conditions.

	ESI	SRM Transition (*m/z*→*m/z*)		Collision	~tR
Mycotoxin	(+ or -)	Quantitative	Qualitative	Energy	(min)
DON	-	341.0→265.0	341.0→247.0	18	6.1
HT2	+	442.2→263.0	442.2→215.0	18	8.9
T2	+	484.2→185.0	484.2→305.0	18	9.3
ZON	-	317.0→174.5	317.0→130.5	25	10.1
FB_1_	+	722.5→334.4	722.5→352.2	40	9.2
OTA	+	404.0→239.0	404.0→358.4	20	9.9
FB_2_	+	706.4→336.2	706.4→354.2	35	10.2
Meloxicam (IS)	+	-	352.0→115.0	20	8.6/9.1

ESI: Electrospray ionization; SRM: selected reaction monitoring; tR: time of retention.

##### 2.2.2.2. Aflatoxins

Aflatoxins were analyzed by fluorescent detection on an HPLC system as previously described by Barmark and Larsson [12] with some modifications. Briefly, 10 g of sample were extracted by mechanical shaking for 45 min using 50 mL 80% acetonitrile in a conical flask. The content was filtered through a Whatman 5971/2 filter paper. One ml of the extracted solution was gently mixed with 40 mL water and the extract was cleaned by passing through an immunoaffinity column at a flow rate of 1 drop/s. Assisted with light vacuum, the column was cleaned with 10 mL of water and the analytes were eluted using 1 mL of methanol and subsequently 3 mL of water. The eluent was transferred into a screw-capped vial and stored at 4 °C (<24 h) before quantification of aflatoxins.

Eluent C was used as the mobile phase run isocratically at 1.0 mL/min. Injection volume of analyte and standard was 250 µL. Aflatoxins were detected at excitation and emission wavelengths of 365 and 430 nm, respectively, after post column derivatization with potassium bromide (cobra cell 100 µA).

##### 2.2.2.3. Method Performance and Quantification of Mycotoxins

Quantification of each analyte was performed by external calibration comparing the peak area to the calibration plot of the corresponding mycotoxin standard. For each set of extraction the apparent recoveries of the mycotoxins, including both the yield of the extraction step and the matrix effects, were determined by spiking known amounts of the mycotoxins to one of the samples and to a blank feed. Hence, 10 g of sample was weighed and spiked with 100 µL of a known concentration of mycotoxin reference standard, thoroughly mixed and kept at room temperature in a fume cupboard for at least 30 min to allow for solvent evaporation. The apparent recovery for each mycotoxin was calculated as: % Recovery = [(A−B)/C] × 100 where A and B are the concentrations of the toxin in spiked and non-spiked samples, respectively, while C is the concentration of the toxin spiked. The recovery data is presented in Table 3 together with the limits of detection (LOD) and quantification (LOQ, calculated as 3 and 10 times, respectively, the noise in the quantitative SRM trace in a blank sample) and the expanded measurement uncertainity (U calculated as 2 times the standard deviation divided by the mean concentration) of the method. Quantification of each analyte was performed by external calibration comparing the peak area to the calibration plot of the corresponding mycotoxin standard. 

**Table 3:** Method performance parameters for the detection and quantification of different mycotoxins.

Mycotoxin	Spiking mycotoxins levels(µg/kg) ^a^	Apparent Recovery ^b^ (%)	LOD (µg/kg)	LOQ (µg/kg)	Expanded Measurement Uncertainty ^c^ (%)
**DON**	300	106 ± 5.6	72	115	30
**ZON**	30.0	127 ± 26.0	3.5	5.2	40
**T2**	40.0	149 ± 27.2	1.0	2.5	17
**HT2**	40.0	167 ± 17.3	2.5	8.0	18
**FB_1_**	100	104 ± 9.5	9.0	30	32
**FB_2_**	100	113 ± 13.4	30	53	30
**OTA**	5.20	145 ± 14.5	2.7	4.6	33
**AFB_1_**	2.70	94 ± 14.5	0.10	0.20	32
**AFB_2_**	1.20	104 ± 11.5	0.10	0.20	32
**AFG_1_**	2.10	100 ± 21.8	0.10	0.20	32
**AFG_2_**	2.10	105 ± 10.9	0.10	0.20	32

^a^ For the LC-MS/MS and HPLC methods, respectively, 10 g of sample was spiked with 100 µL mycotoxin standard solution.

^b^ Mean ± standard deviation.

^c^ Coverage factor of 2, Level of confidence 95% according to Tevell Åberg *et al*. [10].

## 3. Results and Discussion

**Figure 1:**
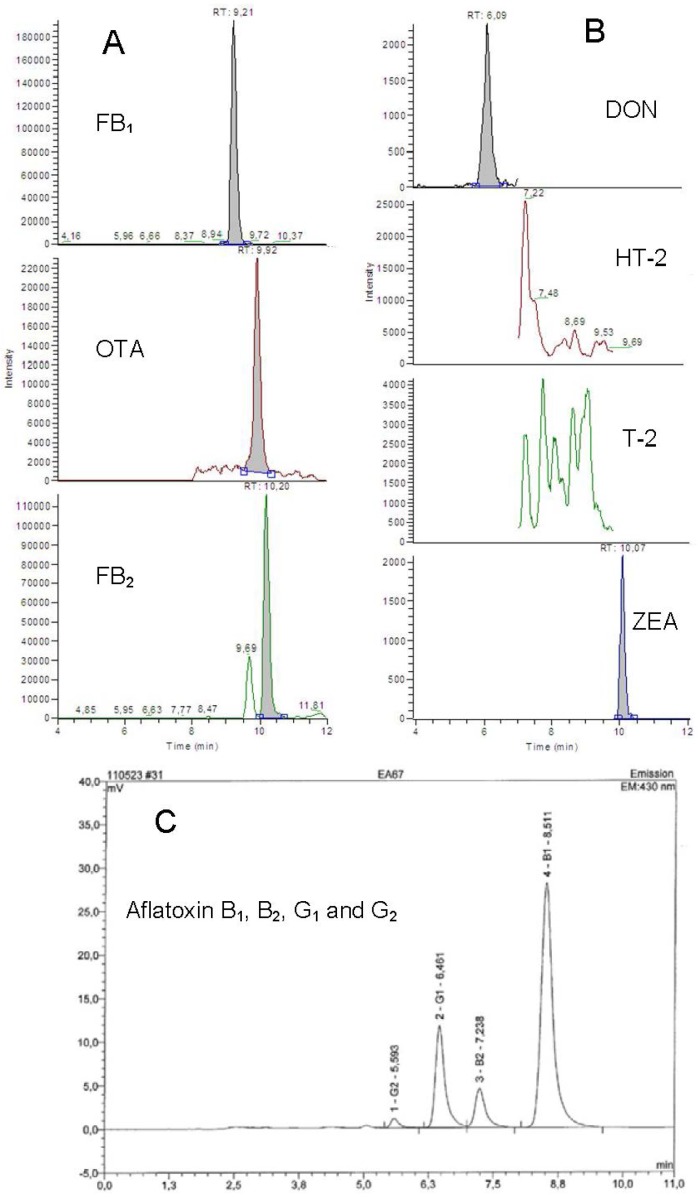
Chromatograms of one of the cattle feed samples analyzed. **A** and **B**: Selected Reaction Monitoring (SRM) chromatograms from the liquid chromatography tandem mass spectrometry (LC-MS/MS) analysis, the sample contained 1800, 17, 520, 850 and 60 µg/kg of FB_1_, OTA, FB_2_, DON and ZON, respectively. T-2 or HT-2 toxin was not detected. **C**: Chromatogram from the HPLC-fluorescence analysis, the sample contained aflatoxins B_1_, B_2_, G_1_ and G_2_ at concentrations of 41.8, 3.4, 25.4 and 2.3 µg/kg, respectively.

In this study, 92 feed samples for different classes of animals were analyzed for AFB_1_, B_2_, G_1_ and G_2_, FB_1_, FB_2_, OTA, DON, ZEA, T-2, and HT-2. The present study is the first survey on multi-mycotoxin occurrence in feeds from S. Africa using an LC-MS/MS. A summary of the prevalence of mycotoxins and associated levels (unadjusted data) detected is presented in Table 4, and chromatograms illustrating a cattle feed sample positive for FB, OTA, DON, ZEA and AFs is presented in Figure 1. The mycotoxin contamination profile clearly demonstrates that AF, FB, OTA, DON and ZEA are common contaminants of S. African compound feeds. All analyzed feed samples were contaminated with mycotoxins, singly or in combination and in order of predominance, FB (100%), DON and ZEA each recovered from 99% of samples, AF and OTA were found in 30% and 4% samples, respectively, meanwhile HT-2 and T-2 were not detected in any of these samples. Except for ZEA and DON, cattle feeds were generally more heavily contaminated (mean levels) with mycotoxins than feeds destined for the other animal species.

**Table 4:** Mycotoxin levels (µg/kg) in compound feeds from South Africa.

		Mycotoxins contamination ^a^														
		AF			FB			OTA			DON				ZEA	
Feed	N ^b^	N + ve ^c^	Mean ^d^	Max	N + ve ^c^	Mean ^d^	Max	N + ve ^c^	Mean ^d^	Max	N +ve^c^	Mean ^d^	Max	N + ve ^c^	Mean ^d^	Max
Chicken	62	14 (8)	0.7 ± 0.7	1.8	62 (54)	903 ± 757	2999	0 (0)	-	-	62 (35)	620 ± 386	1980	62 (24)	100 ± 125	610
Cattle	25	13 (13)	14.7 ± 22.8	71.8	25 (23)	975 ± 686	2497	4 (4)	9.9 ± 4.9	17.1	24 (24)	891 ± 512	2280	24 (19)	72 ± 43	123
Horse	3	1 (1)	0.4 ± 0	0.4	3 (3)	693 ± 617	1338	0 (0)	-	-	3 (3)	742 ± 338	1133	3 (2)	43 ± 5	46
Swine	2	0 (0)	-	-	2 (1)	313 ± 0	313	0 (0)	-	-	2 (2)	1,875 ± 713	2352	2 (2)	148 ± 30	170
**Total**	**92**	**28 (22)**	**9.0 ± 18.7**	**71.8**	**92 (81)**	**916 ± 727**	**2999**	**4 (4)**	**9.9 ± 4.9**	**17.1**	**91 (64)**	**696 ± 490**	**2352**	**91 (47)**	**88 ± 95**	**610**

^a^ T-2 and HT-2 were analyzed but not detected in any of the analyzed samples.

^b^ Number of samples analyzed.

^c^ Number of samples positive with mycotoxins above LOD [LOQ in parentheses].

^d^ Mean levels of mycotoxin contents in positive samples above LOQ expressed as mean ± SD (standard deviation); levels of mycotoxins are not adjusted based on recovery.

South African regulatory limits for mycotoxins in feeds [13]: AF is10 µg/kg for chicks, calves and piglets, 20 µg/kg for poultry and swine, 5 and 50 µg/kg, respectively for dairy and beef cattle; FB is 50,000 µg/kg for cattle and chicken, respectively and 1000 µg/kg for swine; OTA is 20 and 50 µg/kg for chicken and swine; ZEA is 20, 300 and 500 µg/kg for dairy/calves, piglets and swine, respectively; and DON is 1000 µg/kg for swine, 2000, 3000 and 5000 µg/kg, respectively, for calves, dairy and beef cattle, 4000 µg/kg for chickens.

Fumonisins were the most dominant mycotoxins (max: 2999 µg/kg; mean: 916 ± 727 µg/kg) being present in all samples. Since 1988 when fumonisins were discovered in S. Africa [14], they have been recognized as the main mycotoxin contaminant in maize and maize-based products including compound feeds. For compound feeds, 11,000 µg/kg is so far the highest contaminated level reported in S. Africa [15]. Subsequently, surveys of S. African commercial feeds for FB have shown maximum contamination levels below 6000 µg/kg [16,17,18,19].

Concentrations of FB_1_ were comparatively higher, ranging from 104 to 2371 µg/kg compared to FB_2_ where the range was 103 to 673 µg/kg. Higher contamination levels of FB_1_ compared to FB_2_ in cereal grains and animal feeds are often reported [19,20,21]. Levels of fumonisins were generally found to be slightly higher in cattle feeds than in chicken, swine or horse feeds. This variation could not be directly linked to the different levels of maize included since cattle feed contains a much smaller amount of maize than chicken or swine feed. In the present study all samples contained FB below regulatory levels for animal feed in S. Africa. 

Ochratoxin A was the least predominant mycotoxin being present at detectable levels only in cattle feeds with 4 of the 25 samples containing OTA (mean: 9.9 ± 4.9 µg/kg; max. level: 17.1 µg/kg), whereas no OTA was found in feeds for other animals tested (Table 4). Very limited data on OTA contamination of South African animal feeds and feed components is available. In previous surveys on raw materials or finished feeds, neither OTA was found [16,17] nor was it analyzed at all [19]. In the present study, the incidence and levels of OTA were low, however, contrary to the recent study of Stoev *et al.* [18], finished feeds from S. African swine farms with mycotoxic nephropathy were contaminated with OTA at a much higher incidence rate and in several cases, levels above acceptable limits were found. It therefore follows that OTA seems to be a mycotoxin that could be detected in S. African cattle feeds and regularly in swine feeds. The same mycotoxin has been shown to be a nephrotoxin playing a role supposedly in association with other mycotoxins in the aetiology of mycotoxic nephropathy encountered in some farm animals in S. Africa [18] and in Northern and Eastern Europe [22,23,24]. It is also carcinogenic and has some immunotoxic properties [25,26] thus, increasing animal’s susceptibility to secondary infection. In the country, OTA is regulated in feeds for swine and poultry only but no specific limits are yet to be set for other animals including cattle. 

ZEA was present in 99% of the samples and quantification levels were reached in 47 samples (51%) ranging between 30 and 610 µg/kg. Swine feeds had the highest levels of ZEA (range: 127–170 µg/kg) followed by chicken feeds where the levels ranged from 30 to 610 µg/kg. In horse feeds, the range was 39–46 µg/kg. Generally, we found that the distribution pattern of ZEA was similar and highly correlated with that of DON except that DON levels were generally higher in the respective samples. Such a similarity can best be explained based on the fact that these toxins are produced by the same fungi principally *Fusarium graminearum* and *Fusarium culmorum* and that the requirements for production [27,28] are similar. 

Deoxynivalenol is one of the mycotoxins that have received considerable interest in S. Africa [29] as incidences of high DON contamination levels have been reported in agricultural commodities [18,20,30,31]. According to the Southern African Grain Laboratory [30], there are contamination levels in the range of 200–13,000 µg/kg found in maize. Griessler [17] found that 90% of raw materials and compound feed samples contained very high concentrations (mean: 1015 µg/kg; max. of 11,022 µg/kg) of the toxin. Our data revealed that the two swine feeds analyzed had the highest DON contamination levels (1343 and 2356 µg/kg) that were above regulatory limit of 1000 µg/kg for S. Africa. The lowest contamination level of 620 ± 386 µg/kg for DON was recovered from feeds for chicken, while slightly higher mean levels of 742 ± 338 and 891 ± 512 µg/kg were obtained from horse and cattle feeds, respectively. 

Aflatoxin was present in 30% of the samples. As seen in Table 4, cattle feeds (incidence rate of 52%) were the most contaminated with AF (mean: 14.7±22.8 µg/kg; max: 71.8 µg/kg) and 4 of the 25 samples analyzed had levels that exceeded regulatory limits set for South African cattle feeds. Two of these samples were dairy feeds that had levels that were at least 7 times above the limit of 5 µg/kg regulated. The presence of AF particularly AFB_1_ in dairy feed is particularly worrying as levels of 10 µg/kg or even less may result in the appearance of aflatoxin M_1_ (AFM_1_) in the milk at levels above those now permissible (0.05 µg/kg) in Europe or South Africa [32]. Poultry and horse feeds also contained AF, but contamination levels were comparatively low (max level: 1.8 µg/kg or below) with none detected in swine feeds. AFB_1_ and AFG_1_ occurred more frequently and at higher levels than AFB_2_ and AFG_2_. However, in 3 samples, AFG_2_ was the only AF detected either singly or together with AFG_1_. 

In our study, a quantitative difference in mycotoxin contamination between the different feed types was evident and as previously stated, cattle feeds were the most contaminated. Aflatoxin levels were higher in cattle feeds, *i.e.*, having mean levels >20 times higher than the levels recovered from other types of feed and in particular, OTA was only detected in feeds for cattle (Table 4). The most likely reason for this variation may be the use of different feed raw materials for different types of animals and also the level of inclusion (Table 1). Breytenbach, L. in an email [33] stated that in S. Africa, cotton seed oilcake or full fat cotton seed is directed solely for ruminant feed production and the same feed ingredients were consistently found by Reiter *et al*. [10] and Mngadi *et al*. [16] to contain high levels of AF. Those raw materials may also contain OTA probably why high levels of these toxins were observed in some cattle feeds.

Mngadi *et al.* [16] and Stoev *et al.* [18] provided some data in which several mycotoxins co-occur in feeds within the country. In our study, the co-existence of mycotoxins within the same sample was very common and data revealed that 91 samples contained multiple mycotoxins, with 67% that with three mycotoxins (FB + DON + ZEA), 26% with 4 mycotoxins (FB + DON + ZEA + AF), while 5 samples had 5 mycotoxins (FB + DON + ZEA + AF + OTA). Based on the S. African feed regulations, 13% of samples, mostly for cattle, contained mycotoxin levels regarded as unfit for livestock feeding. Accordingly, 7 feed samples showed unacceptable levels of ZEA and AF and in 4 of these (calf and dairy feeds), co-contamination of ZEA and AF occurred at unacceptable levels. In addition some cattle feed samples also contained extremely high AF levels and the same samples also contained high amounts of OTA. 

Such multiple mycotoxin contaminations in feeds is crucial as mycotoxins may toxicologically interact with each other eliciting marked synergistic and additive actions [32] especially between mycotoxins found at high concentrations. These actions provoked by OTA at low levels combined with FB_1_, have been the prime suspect for increased incidence of mycotoxic nephropathy in S. African swine [18]. This theory was experimentally tested [34] when swine were fed diets containing OTA and/or FB_1_ and the same mycotoxic nephropathy observed under field conditions was enhanced in animals exposed to these mycotoxins in combination. 

## 4. Conclusions

From the results of this screening of AFB_1_, B_2_, G_1_ and G_2_, FB_1_, FB_2_, OTA, DON, ZEA, T-2, and HT-2 in various kinds of feed, it can be concluded that in South Africa, cattle feeds are probably more contaminated with mycotoxins than feeds for other animal species as they were found to contain the highest mean levels of AF and FB. In addition, OTA contamination only occurred in cattle feeds and in most of the positive samples, this toxin co-existed with other mycotoxins at high contamination levels that could induce some synergistic or additive effects in animals. Because such mycotoxins as AF and OTA can be carried over to animal-derived food products, human exposure to such mycotoxins may be a problem. Feed raw materials and finished feeds should for that reason be routinely monitored in order to identify potential sources of mycotoxins. 
